# Cognitive performance in children and adolescents at high-risk for obsessive-compulsive disorder

**DOI:** 10.1186/s12888-020-02751-5

**Published:** 2020-07-20

**Authors:** Elisa Teixeira Bernardes, Leonardo Cardoso Saraiva, Marina de Marco e Souza, Marcelo Queiroz Hoexter, Priscila Chacon, Guaraci Requena, Euripedes Constantino Miguel, Roseli Gedanke Shavitt, Guilherme Vanoni Polanczyk, Carolina Cappi, Marcelo Camargo Batistuzzo

**Affiliations:** 1 Departamento de Psiquiatria, Hospital das Clinicas HCFMUSP, Faculdade de Medicina, Universidade de Sao Paulo, R. Dr Ovidio Pires de Campos, 875, Sao Paulo, SP Brazil; 2 Instituto de Ciencias Exatas e Tecnologicas da Universidade Federal de Vicosa, Viçosa, Brazil; 3 Curso de Psicologia, Faculdade de Ciências Humanas e da Saúde, Pontifícia Universidade Católica de São Paulo, Sao Paulo, SP Brazil

**Keywords:** Obsessive-compulsive symptoms, High-risk, Obsessive-compulsive disorder, First degree relatives, Neuropsychological assessment, Cognitive functions

## Abstract

**Background:**

Cognitive performance has been studied in adults with obsessive-compulsive symptoms (OCS) and in adult relatives of patients with obsessive-compulsive disorder (OCD) Meanwhile, few studies have been conducted with children under the same conditions. This study compared the neurocognitive domains previously associated with dysfunction in OCD, especially visuoconstructive ability, visuospatial memory, executive functions, and intelligence, in children and adolescents at high risk (HR) for OCD (*n* = 18) and non-OCD controls (NOC) (*n* = 31).

**Methods:**

For the HR group, we considered the first-degree relatives of patients with OCD that present OCS, but do not meet diagnostic criteria for OCD. Psychiatric diagnosis was assessed by experienced clinicians using the Structured Clinical Interview for DSM-IV and OCS severity was measured by the Yale-Brown Obsessive-Compulsive Scale. Neurocognitive assessment was performed with a comprehensive neuropsychological battery. Performance on the cognitive domains was compared between groups using Multivariate Analysis of Variance, whereas performance on the neuropsychological variables was compared between groups using independent t-tests in a cognitive subdomain analysis.

**Results:**

The cognitive domain analysis revealed a trend towards significance for impairments in the motor and processing speed domain (*p* = 0.019; F = 3.12) in the HR group. Moreover, the cognitive subdomain analysis identified a statistically significant underperformance in spatial working memory in the HR group when compared to the NOC group (*p* = 0.005; *t* = − 2.94), and a trend towards significance for impairments in non-verbal memory and visuoconstructive tasks in the HR group.

**Conclusions:**

Our results suggest impairments in spatial working memory and motor and processing speed in a non-clinical sample of HR participants. Considering the preliminary nature of our findings, further studies investigating these neurocognitive domains as potential predictors of pediatric OCD are warranted.

## Background

Obsessive-compulsive disorder (OCD) is a neuropsychiatric disorder characterized by intrusive thoughts (obsessions) and repetitive behaviors (compulsions) [1]. With a lifetime prevalence of 1.5–2.5% [2, 3], OCD constitutes a common disorder which onset typically occurs during childhood or early adulthood [4] and that presents two peaks of onset, being the first one during preadolescent childhood (around eleven  years) and the other around late adolescence and early adulthood [5]. Current pharmacological and psychotherapeutic treatments can benefit 60–70% of patients [6, 7] and treatment-refractory disease is common [8, 9]. In addition, untreated OCD usually persists and becomes chronic [10]. As such, further investigations are warranted for advances in OCD care and prevention.

Consistent with its genetic underpinnings [11], several family studies have demonstrated that first-degree relatives (FDRs) of individuals with OCD are at an increased risk for developing the disorder [12, 13]. In addition, diverse genetic approaches have indicated that subclinical OCD and the full-blown disorder share a considerably similar genetic predisposition [14, 15]. Since subclinical OCD can portend the full-blown disorder in children and adolescents, especially those with greater genetic susceptibility [16], the discovery of markers of vulnerability to OCD in this high-risk population could lead to the development of novel approaches for early detection and management of susceptibility to OCD in the pediatric population. Such approaches would, therefore, improve OCD prevention.

Impairment in multiple cognitive functions has been consistently reported among patients with OCD [17]. Underperformance in the intelligence quotient (IQ) has been demonstrated in adult OCD, with more severe deficits in performance IQ as compared to verbal IQ [18]. Moreover, deficits in visuospatial abilities, executive functions, verbal memory, verbal fluency, and attention have been reported in adults with OCD [19]. Given the genetic nature of the disorder, putative impairment in several cognitive functions has been extensively investigated in unaffected FDRs of individuals with OCD [20–27]. Consistently, deficits in inhibitory control [21, 22], decision making [23, 24], long-term verbal and visual memories [25], planning [26], working memory, verbal fluency and motor speed [27] have been found in adult FDRs of individuals with OCD. Considering the contribution of genetic factors in the etiology of early-onset OCD [28], impaired inhibitory control and cognitive flexibility were recently reported for an adult sample of early-onset OCD patients and their unaffected FDRs [29].

Cognitive function has been less extensively investigated in pediatric OCD when compared to adult OCD [30], which could account for the inconsistency of findings reported in previous studies. Indeed, a meta-analysis revealed no significant impairments in cognitive functions associated with pediatric OCD, possibly due to the small number of studies included [31]. Conversely, deficits in visual memory, visual organization, processing speed, cognitive flexibility, and planning have been reported for pediatric OCD [30, 32–34]. Moreover, the assessment of cognitive function in pediatric FDRs of individuals with OCD has been largely unexplored. To our knowledge, only one study so far has assessed the cognitive performance of pediatric patients with OCD, their unaffected FDRs, and healthy individuals [34]. Both patients with OCD and their FDRs exhibited underperformance in planning tasks. Such findings warrant further investigations of the cognitive function in pediatric FDRs of individuals with OCD.

Furthermore, research has been conducted on the association between cognitive dysfunction and subclinical OCD. Previous investigations have not found neuropsychological deficits among adults with obsessive-compulsive symptoms (OCS) [35, 36], notwithstanding preliminary evidence supports an association between cognitive function and OCS among children. For instance, response inhibition and set-shifting have been shown to predict OCS in children younger than six-years-old, and response inhibition has been shown to predict OCS in children older than six-years-old [37]. Such findings suggest that cognitive impairment is associated with subclinical pediatric OCD. Considering the combined evidence of cognitive impairment in pediatric FDRs of individuals with OCD and in pediatric individuals manifesting subclinical OCD, the investigation of cognitive dysfunction as a marker of vulnerability to OCD in children and adolescents may enhance OCD prevention.

Therefore, in the present study, our aim was to assess the cognitive performance of pediatric individuals at high risk (HR) for OCD in comparison to non-OCD controls (NOC). Accordingly, we defined the presence of subclinical OCD and being an FDR of a patient with OCD as the criteria for the inclusion of pediatric participants in the HR group. To our knowledge, the present study is the first to investigate cognitive functioning in pediatric FDRs with subclinical OCD.

## Method

### Design and recruitment

As part of the ongoing projects conducted by the National Institute of Developmental Psychiatry for Children and Adolescents [14, 38], the present cross-sectional study was conducted in a pediatric sample of 18 HR and 31 NOC (see flowchart in the supplementary files for details). The participants were recruited through media advertisements and an active search conducted at private and public schools. The inclusion criteria for the HR group were: 1) age between 7 and 18 years; 2) being a first-degree relative (sibling or offspring) of an individual with OCD; 3) presenting OCS; 4) not meeting diagnostic criteria for OCD and 5) not having undergone or currently undergoing any sort of psychiatric treatment. The exclusion criteria for the HR group were: 1) history of head injury; 2) history of substance abuse; 3) presence of intellectual disability or any other neuropsychiatric diagnosis; 4) the presence of any neurological condition and 5) pregnancy or lactation. Apart from being a first-degree relative of an individual with OCD and presenting OCS, the inclusion and exclusion criteria for the NOC group were similar to the aforementioned criteria.

Diagnostic assessments to confirm OCD status and assess other comorbidities were conducted by experienced clinicians using the Structured Clinical Interview for DSM-IV (SCID-I) [39]. Once the OCD participants were confirmed to meet study criteria and after permission, we contacted the first relatives (siblings and offspring) of the participants. For assessment of psychiatric diagnosis among the FDRs, the SCID-I was administered for adults and the Kiddie Schedule for Affective Disorders and Schizophrenia [40] was administered for pediatric individuals. The Yale-Brown Obsessive-Compulsive Scale (Y-BOCS) [41] was used for measuring the severity of OCS. Additionally, the Petersen Puberty Scale [42] and the Edinburgh Handedness Inventory [43] were administered to ascertain the pubertal status of pediatric participants and their handedness, respectively.

### Neuropsychological assessment

The neuropsychological battery comprised tests that assessed the following cognitive domains: intelligence, attention, motor and processing speed, visuoconstructive abilities, verbal and visuospatial memories, working memory, cognitive flexibility, and inhibitory control. The neuropsychological tests administered and cognitive domains are outlined in Tables 1 and 2, respectively. The neuropsychological tests were administered by experienced psychologists in sessions which duration on average lasted ninety minutes. No issues in terms of fatigue or cooperation from the part of the participants were reported for those sessions.

**Table 1:** Neuropsychological Tests Administered in the Study

Name	Definition
**Wechsler Abbreviated Scale of Intelligence (WASI)** [44, 45]	Estimates a total IQ score, which is further partitioned into verbal and performance IQ scores. Verbal IQ: Vocabulary: the participant needs to define the meaning of certain words. Similarities: the participant must ascertain the similarities between two words. Performance IQ: Block Design: requires the participant to assemble colored blocks into two-color figures. Matrix Reasoning: the participant has to complete a geometric pattern by selecting the best-fitting picture out of a set of pictures.
**Rey Auditory Verbal Learning Test (RAVLT)** [46]	A word-list with 15 items repeated 5 times. In each repetition, the subject needs to recall the maximum number of words. There is also an interference 15-word list and a delayed recall, after 30 min.
**Trail-Making Test (TMT)** – [47]	This paradigm comprises five conditions: 1) visual scanning: visual cancellation task. 2) number sequencing: connect the dots, using a numerical sequence. 3) letter sequencing: connect the letters, in an alphabetical sequence. 4) number-letter switching: connect dots, using numbers and letters in a numerical and alphabetical sequence. 5) motor speed: connect filled circles based on a dotted trail.
**Design Fluency Test (DFT) -** [47]	This test comprises three conditions, in each the participant needs to create as many designs as possible in a limited time: 1) Filled dots: connecting filled/black dots. 2) Empty dots: connecting empty/white dots. 3) Switching: connecting filled and empty dots.
**Color and Word Interference Test (CWIT) -** [47]	This task comprises four conditions, which need to be completed as quickly as possible: 1) Condition 1: A set of colors is shown and the participant needs to name the colors. 2) Condition 2: A set of names of colors printed in black are shown and the participant needs to read the words. 3) Condition 3: A set of color names is printed in different colors (ex: “blue” printed in pink) and the participant needs to inhibit the tendency to read the words, and then voice the names of the colors in which the words are printed. 4) Condition 4: A set of colors names is printed outside and inside a rectangle and the participant needs to read the names of the colors when they are inside the rectangle and to voice the colors in which the words are printed when they are outside a rectangle.
**Wisconsin Card Sorting Test (WCST)** [48]	WCST is a classic executive functioning test in which the subject has to combine cards following a specific rule that he does not know (color, geometric form, or number) and for each trial, the subject receives feedback saying if the match is right or wrong.
**Go/NoGo task** [49]	It consists of a computerized test, built on a homemade paradigm (E-Prime) The participant has to press the spacebar whenever a letter can be seen on the computer screen, as quickly as possible (condition “Go”). Alternatively, during the “NoGo” conditions, the participant was required to press different keyboard keys according to the colors of a letter presented on a computer screen. The condition “NoGo” is composed of specific letters in specific colors (‘O’ in blue or ‘E’ in pink). There are a total of 96 trials: 72 “Go” and 24 “NoGo”.
**Grooved Pegboard Task** [50]	This test comprises two conditions, in each the participant has to use only one hand to fill twenty-five holes with pegs in a predetermined order, as quickly as possible.
**Rey-Osterrieth Complex Figure (ROCF)** [51]	It consists of a visuospatial task in which the subject needs to copy a complex and detailed geometrical figure, and recall it without seeing it again, after 3 and after 30 min.
**Corsi Block-Tapping Test (CBTT)** – Wechsler Memory Scale (WMS-R) [52]	This test comprises forward and backward conditions. In the forward condition, the participant observes the examiner taping a set of blocks in a particular sequence, and then is required to tap the blocks in the exact same sequence. The backward condition follows a similar paradigm, with the exception that the participant is required to tap the blocks in the inverse sequence.
**Digit Span Test (DST)** – Wechsler Intelligence Scale for Children – Third Edition [53]	It consists of a verbal paradigm which also comprises forward and backward conditions. In the DST, the participant is required to listen to a numeric sequence and repeat the sequence in the same and inverse orders during the forward and backward conditions, respectively.
**Brixton Test** - [54]	It requires the participant to predict the position of a circle based on its previous positions.

**Table 2:** Neuropsychological Domains and Tests Evaluated in the Study

Domain Definition	Subtest variables
**Intelligence** General intelligence (IQ)	Wechsler abbreviated scale of intelligence (WASI) [44, 45] Block Design; Matrix; Vocabulary; and Similarities.
**Attention** Endogenous processing of selecting relevant stimuli (concentrating) in the environment (e.g., objects)^a^	Rey auditory verbal learning test (RAVLT) [46] Span A; Span B
	Trail making test (TMT) – Delis-Kaplan executive function scale (D-KEFS) [47] 1^st^ condition omissions; and 4^th^ condition sequence errors
	Design fluency test (DFT) – D-KEFS [47] DFT 1 and 2 - % errors
	Wisconsin card sorting test (WCST) [48] WCST failures to maintain set.
	Go/NoGo [49] Go/NoGo omissions.
**Motor and processing speed** Ability to quickly process information and execute it (fine motor skills)	Color-word interference test (CWIT) - D-KEFS [47] Color naming time (CWIT 1); and word reading time (CWIT 2)
	Grooved pegboard test [50] Dominant hand time; and non-dominant hand time
	TMT – D-KEFS [47] 5^th^ condition time
**Visuoconstructive abilities** Coordination of fine motor skills with spatial abilities	Block Design test - WASI [44, 45]
	Rey-Osterrieth complex figure (ROCF) [51] Copy total score
**Visuospatial memory** Memory for visual and spatial information	Corsi block-tapping test (CBTT) - Wechsler Memory Scale (WMS-R) [52] Forward hits
	ROCF [51] Immediate recall; and Delayed recall
**Verbal memory** Memory for verbal information	Digit span test (DST) - Wechsler Intelligence Scale for Children (WISC-III) [53] Forward hits
	Rey auditory verbal learning test (RAVLT) [46] Immediate recall; and delayed recall
**Working memory** Ability to retain information and perform mental operations from them	DST – WISC-III [53] Backward hits
	CBTT – WMS-R [52] Backward hits
**Cognitive flexibility** Ability to change the perspectives, thinking of new possibilities for solving a problem	WCST [48] Perseverative errors; and categories DFT – D-KEFS [47] DFT 3 - % perseverative responses
	Brixton [54] Brixton hits
	TMT – D-KEFS [47] 4^th^ - 5^th^ condition time difference
**Inhibitory control** Ability to resist an inclination to perform an action and, opting for a more convenient one	Go/NoGo – commission errors
	CWIT – D-KEFS [47] CWIT 3 errors; CWIT 4 errors; and CWIT 3-1 time difference

^a^ Attention is not a unitary system – it refers to several different capacities of how the organism comes receptive to stimuli and the ability to maintain concentration focused on stimuli over time (sustained attention). WASI – Wechsler abbreviated scale of intelligence; TMT – Trail making test; D-KEFS – Delis-Kaplan executive function scale, DTF – Design fluency test; WCST – Wisconsin card sorting test; CWIT – Color-word interference test; CBTT – Corsi block-tapping test; WMS – Wechsler Memory Scale; ROCF – Rey-Osterrieth complex figure; DST – Digit span test; WISC III – Wechsler Intelligence Scale for Children 3rd edition; RAVLT – Rey auditory verbal learning test

### Ethical considerations

This study was approved by the Medical Ethics Committee of the Faculty of Medicine at the University of Sao Paulo. All participants and their respective parents or legal guardians were informed about the procedures pertaining to the study and provided their written informed consent prior to enrollment of the participants in the study.

### Data analysis

Demographic and clinical variables were analyzed using the independent samples t-test for continuous variables and the Chi-squared test for categorical variables. Normality assumptions of neuropsychological variables were evaluated using the Shapiro-Wilk test. Violations of the normality assumptions were assessed according to the statistical significance threshold (*p*-value) set at 0.01. In the cases of non-normal distribution, a correction was applied using the ‘bestNormalize’ function of RStudio (package `bestNormalize`). After that, the normality of the variables was reassessed.

The neuropsychological variables were analyzed at the domain and the subdomain levels. In an initial analysis at the domain level, neuropsychological variables were grouped as follows: intelligence, attention, motor and processing speed, visuoconstructive abilities, visuospatial memory, verbal memory, working memory, cognitive flexibility, and inhibitory control. The global performance on each cognitive domain was compared between the groups using the Multivariate Analysis of Variance (MANOVA) (R package ‘stats’, function ‘manova’). Moreover, a further analysis was conducted at the subdomain level, whereby performance on the neuropsychological variables within each domain was compared between the groups using the independent samples t-test (R package ‘stats’, function ‘t.test’). Additionally, effect sizes (Cohen’s d) for each between-group comparison were also computed (R package ‘lsr’, function ‘cohen’). After group comparisons, a post-hoc power calculation analysis was performed for the cognitive subdomain analysis. The Bonferroni correction was applied to all analyses conducted considering the number of cognitive domains assessed, as in Purcell and colleagues [55]. As such, a stricter statistical significance threshold was set at *p* = 0.0055 (0.05/9). Considering the exploratory nature of this study, *p* values between 0.05 and 0.0055 were considered as a trend towards significance, which is referred as nominal significance from this point onwards. Statistical analyses were performed using the RStudio, version 1.2.1335 (2019).

## Results

### Demographic and clinical variables

No statistically differences between the groups were found in terms of sex, pubertal development, handedness, years of education, and total IQ (Table 3). Both groups presented total IQ scores in the normal range. As measured by the Y-BOCS, the severity of OCS in the HR group was below the clinical range.

**Table 3:** Demographic and clinical characteristics of the high-risk and non-OCD control individuals

	High-Risk (n = 18)	Non-OCD Control (*n* = 31)	*p*-value
	n (%) / M (SD)	n (%) / M (SD)	
Sex			
Male	12 (66%)	18 (58%)	0.551 ª
Puberty Development			
Age	11.1 (2.4)	11.7 (1.9)	0.365 ^b^
Handedness			
Right	16 (89%)	30 (96.8%)	0.377 ª
Education Level			
Years of Education	6.1 (2.5)	6.3 (2.1)	0.743 ^b^
Total IQ	103.5 (12.2)	105.6 (13.5)	0.592 ^b^
Y-BOCS			
Total	7.1 (5.3)	–	**–**
Obsessions	3.6 (2.6)	–	**–**
Compulsions	3.5 (2.9)	–	**–**

ª Chi-squared Test \ ^b^ Independent t-test. M – mean; SD – standard deviation; IQ – Intelligence quotient; Y-BOCS – Yale-Brown Obsessive Compulsive Scale

### Cognitive domains analysis

**Fig. 1:**
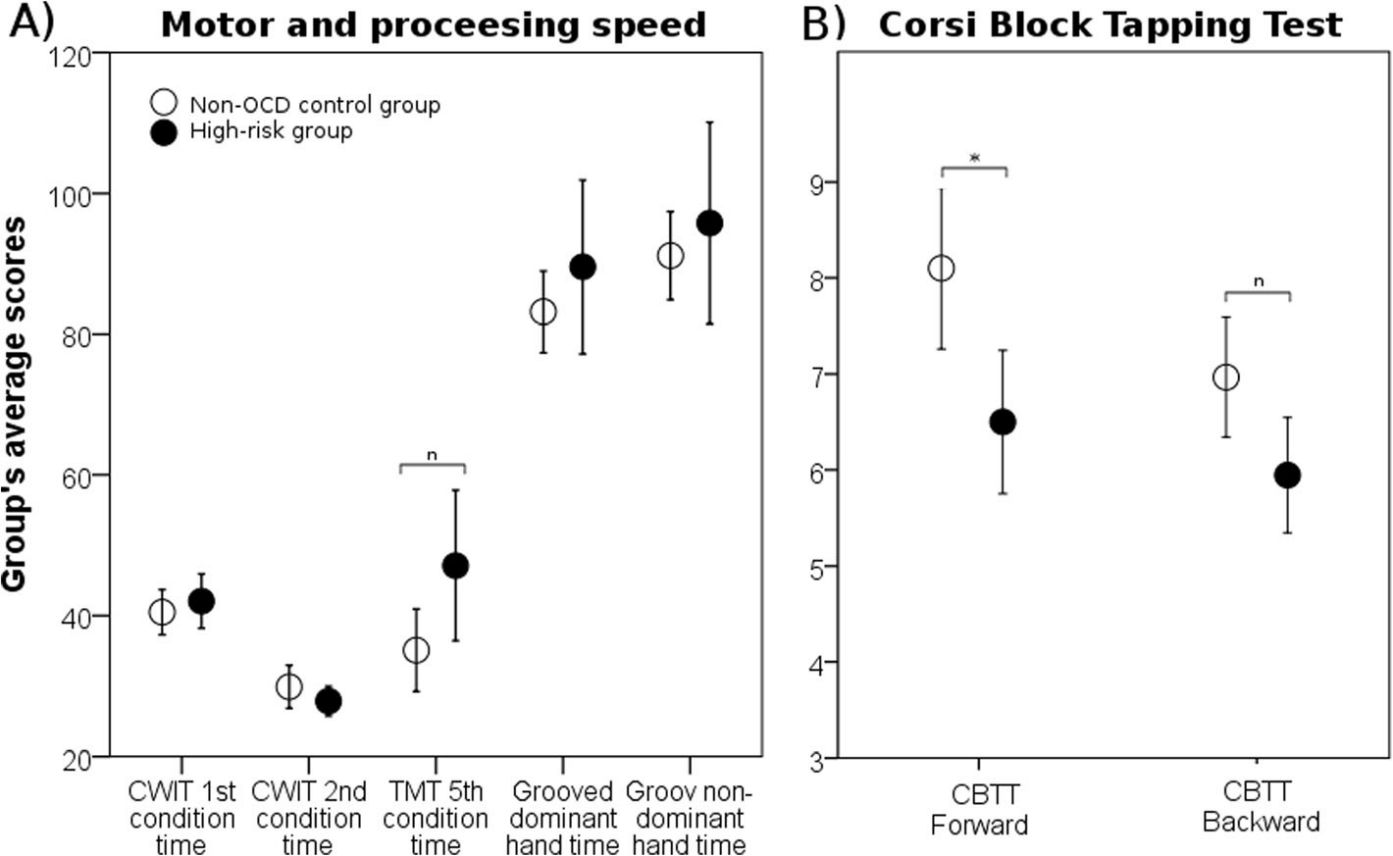
Groups’ average scores for **a**) the motor and processing speed MANOVA (higher punctuation means worse performance), and **b**) total IQ and IQ discrepancy (the difference between verbal IQ and performance IQ). Error bars means a 95% confidence interval (CI). OCD - obsessive-compulsive disorder; CWIT – Color-Word Interference Test; TMT – Trail Making Test; CBTT - Corsi Block Tapping Test. ^n^*p-value* < 0.05 (nominal significance); * *p-value* < 0.0055 (Bonferroni corrected)

The cognitive domain analysis revealed that the HR group exhibited a nominally significant overall underperformance in tasks measuring the motor and processing speed abilities (*p* = 0.019; F = 3.115) (Fig. 1a). No statistically or nominally significant difference in overall performance was found for the other cognitive domains (Table 4). Only the scores in the immediate recall condition of the ROCF paradigm were removed from the cognitive domain analysis due to multicollinearity (r > 0.9).

**Table 4:** Difference Within Neurocognitive Domains

Neuropsychological measure	F	Df	*p-value* MANOVA
Estimated intellectual efficiency	2.085	4	0.099
Attention	0.718	7	0.657
Motor and processing speed	3.115	5	0.019^n^
Visuoconstructive abilities	1.868	2	0.166
Visuospatial memory	2.787	3	0.051
Verbal memory	0.679	3	0.570
Working memory	3.046	2	0.057
Cognitive flexibility	2.362	5	0.057
Inhibitory control	0.325	4	0.859

MANOVA – multivariate analysis of variance; Df – Degree of Freedom; ^n^*p-value* < 0.05 (nominal significance)

### Cognitive subdomain analysis

Means and standard deviations for each of the neuropsychological variables are displayed in Table 5. The cognitive subdomain analysis revealed that the difference between the groups in the number of correct taps in the forward condition of the Corsi Block Tapping Test was statistically significant after the Bonferroni correction [t (45) = − 2.94, *p* = 0.0050, d = 0.79] (Fig. 1b). In addition, group-differences in the following outcome variables achieved nominal significance: the number of correct taps in the backward condition of the Corsi Block Tapping Test [t (44) = − 2.31, *p* = 0.0260, d = 0.67], the time to complete the fifth condition of the Trail Making Test [t (28) = 2.27, *p* = 0.0301, d = 0.69], the scores in the Block Design subtest [t (43) = − 2.08, *p* = 0.042, d = 0.57] and the discrepancy between the scores of verbal IQ and performance IQ [t (29) = 2.11, *p* = 0.043, d = 0.66]. No statistically or nominally significant difference between the groups in other outcome variables was found. A secondary subdomain analysis was performed to compare the neuropsychological variables between the groups adjusting for sex and age, which revealed results in the same direction as those obtained in the comparison using independent t-tests (data not shown). A power analysis conducted after the group comparisons revealed that the cognitive subdomain analysis had 80% power to detect group differences with Cohen`s d = 0.75 and 37.65% power to detect group differences with Cohen`s d = 0.4, in both cases considering alpha = 0.05 (one-tailed).

**Table 5:** Mean, standard deviation, range and between-groups comparison of neuropsychological variables

	High Risk n = 18		Non-OCD Control n = 31			
Neuropsychological measure	Mean	(SD)	Mean	(SD)	*t*-test	*p*-value
IQ						
Total (WASI)	103.5	(12.2)	105.6	(13.4)	0.07	0.938
Verbal (WASI)	112.5	(15.2)	110.5	(15.4)	1.02	0.313
Performance (WASI)	93.9	(9.8)	99.7	(10.1)	−1.28	0.207
Verbal-Performance Discrepancy^a^	18.61	(13.2)	10.8	(11.6)	2.11	0.043^n^
ATTENTION						
RAVLT span A	6.2	(1.5)	6.4	(1.6)	−0.49	0.622
RAVLT span B	5.7	(1.9)	5.6	(1.5)	0.19	0.843
TMT 1st condition omissions	0.2	(0.7)	0.2	(0.4)	−0.08	0.936
TMT 4th condition sequence errors	0.4	(1.0)	0.4	(0.6)	−0.15	0.879
DFT 1 e DFT 2 - %errors	.12	(0.2)	.04	(0.1)	1.47	0.152
WCST failures to maintain set	1.1	(1.2)	1.2	(1.0)	−0.30	0.764
Go-NoGo omissions	3.6	(6.5)	2.6	(4.0)	0.78	0.439
MOTOR AND PROCESSING SPEED						
CWIT color naming time	42.1	(7.8)	39.9	(9.1)	0.86	0.390
CWIT word reading time	27.8	(4.3)	28.9	(7.4)	−0.67	0.503
TMT 5th condition time	47.1	(21.5)	34.0	(14.4)	2.27	0.030^n^
Grooved dominant hand time	89.5	(24.9)	83.7	(15.0)	0.90	0.376
Grooved non-dominant hand time	95.8	(28.9)	92.4	(19.0)	0.44	0.662
VISUOCONSTRUCTIVE ABILITIES						
Block Design Test	19.0	(9.5)	25.6	(12.4)	−2.08	0.042^n^
ROCF total score – copy	28.8	(6.5)	30.5	(3.7)	−0.59	0.558
VISUOSPATIAL MEMORY						
CBTT forward hits	6.5	(1.5)	8.1	(2.3)	−2.94	0.005*
ROCF immediate recall	18.7	(6.7)	19.2	(5.4)	−0.26	0.792
ROCF delayed recall	18.3	(6.8)	18.4	(5.6)	−0.08	0.930
VERBAL MEMORY						
DST forward hits	7.5	(1.3)	7.0	(2.1)	1.15	0.254
RAVLT immediate recall	9.9	(2.2)	10.3	(2.6)	−0.55	0.583
RAVLT delayed recall	9.9	(2.3)	10.3	(2.9)	−0.21	0.828
WORKING MEMORY						
CBTT backward hits	5.9	(1.2)	7.0	(1.7)	−2.31	0.026^n^
DST backward hits	4.8	(1.7)	4.7	(1.7)	0.25	0.797
COGNITIVE FLEXIBILITY						
WCST Perseverative errors	10.4	(3.5)	9.8	(3.8)	0.46	0.642
WCST categories	2.4	(1.2)	2.5	(1.2)	−0.10	0.913
DFT %Perseverative errors	.13	(0.2)	.03	(0.1)	0.74	0.464
TMT 4–5	96.2	(86.3)	84.3	(50.6)	0.15	0.877
Brixton hits	36.6	(8.5)	40.0	(4.1)	−1.16	0.254
INHIBITORY CONTROL						
Go-NoGo Commission errors	8.8	(3.9)	8.5	(3.7)	0.25	0.800
CWIT 3 errors	2.8	(3.5)	2.0	(2.8)	0.64	0.525
CWIT 4 errors	2.0	(2.2)	2.5	(3.4)	−0.17	0.863
CWIT 3–1 time difference	39.1	(18.8)	36.5	(18.0)	0.37	0.709

^a^ The Verbal – Performance difference is calculated by subtracting the performance IQ from the verbal IQ. WASI – Wechsler abbreviated scale of intelligence; RAVLT – Rey auditory verbal learning test; TMT – Trail making test; DFT– Design fluency test; WCST – Wisconsin card sorting test; CWIT – Color-word interference test; ROCF – Rey-Osterrieth complex figure; CBTT – Corsi block-tapping test; DST – Digit span test. ^n^ p-value < 0.05 (nominal significance); * *p-value* < 0.0055 (according to Bonferroni correction)

## Discussion

The purpose of this study was to investigate the cognitive performance of pediatric individuals at HR for OCD in comparison to NOC control pediatric individuals. At the cognitive domain level, our analyses revealed nominally significant motor and processing speed impairments in the HR group as compared to the NOC group. On the other hand, at the subdomain level, we observed spatial working memory deficits in the HR group and nominally significant impairments in non-verbal memory and visuoconstructive tasks in the HR group.

Previous studies evaluating adults with OCD have consistently reported impairments in processing speed [56–61], which have also been reported for adult FDRs of patients with OCD [27]. Likewise, the assessment of neuropsychological function in the largest pediatric sample to date identified significant underperformance in tasks measuring processing speed among patients with OCD, in comparison to individuals who do not have the disorder [30]. Moreover, deficits in this cognitive domain have been associated with ordering and symmetry symptoms manifested by youth with OCD [62]. Since treatment response has been shown to improve the deficits in processing speed among both pediatric [63] and adult patients [64, 65] with OCD, it could be hypothesized that impairments in this cognitive domain represent a modifiable vulnerability marker for OCD across the lifetime. In this sense, a study reported that pathological uncertainty in adult OCD patients underlies deficits in processing speed [66], which suggests that behavioral interventions could improve processing speed skills and consequently benefit children and adolescents at higher risk for the disorder. Consistent with the transdiagnostic etiologies of psychiatric disorders [67, 68], deficits in processing speed have been found in adult patients with schizophrenia and comorbid OCD [69] or OCS [70], suggesting that such impairments may constitute a broader vulnerability marker for related psychiatric disorders.

The cognitive subdomain analysis revealed significant underperformance in spatial (nonverbal) working memory, as measured by the Corsi Block Tapping Test in the pediatric participants at HR for OCD, in comparison to NOC. Associations between pediatric OCD and impairments in nonverbal memory have been inconsistently reported [30–32, 71, 72]. Likewise, the only study, to our knowledge, which investigated neuropsychological dysfunction among pediatric FDRs of patients with OCD found no impairments in spatial working memory [34]. Nonetheless, accumulating evidence supports the association between deficits in nonverbal memory and adult OCD [55, 73–82]. Indeed, comprehensive meta-analyses revealed significant associations between deficits in nonverbal memory and adult OCD [19, 83]. Moreover, a recent meta-analysis indicated that adult FDRs of patients with OCD exhibit impairments in short-term visuospatial memory [84].

Moreover, the cognitive subdomains analysis revealed a nominally significant discrepancy between higher verbal and lower performance IQ scores among pediatric participants at HR for OCD, as compared to NOC. Supporting the impairment in processing speed detected in the cognitive domain analysis, a nominally significant difference between groups was found in the time to complete the fifth condition of the Trail Making Test. In accordance with these findings, a recent study identified a significant discrepancy between higher verbal and lower performance IQ scores in pediatric OCD patients, as compared to pediatric healthy developing individuals [89]. Those findings are consistent with a recent meta-analysis indicating a discrepancy between higher verbal and lower performance IQ scores in adult patients with OCD [18], which could be explained by their poorer processing speed negatively affecting the performance IQ scores [18, 85, 86]. Previous investigations have indicated that such discrepancy is associated with reduced motor competence among preschoolers [87] and functional neuroimaging-detected alterations during cognitive conflict resolution among children and adolescents [88]. In this regard, the appropriate school environment has reportedly contributed to improvements in the discrepancy between verbal and performance IQ scores [90] (Lapierre et al., 1992). Further investigations are warranted to confirm this discrepancy in children and adolescents at HR for OCD, which could foster early interventions in the course of the disease. 

The major limitation of the current study is the small sample size, which limits the detection of significant differences between the groups. Therefore, the reported findings should be considered preliminary, requiring further confirmation in larger samples. Nonetheless, the present study has raised pertinent hypotheses that are well integrated into the existing literature on the topic. Indeed, one longitudinal study reported that impairments in motor and visuospatial skills predict the maintenance of OCD from childhood into adulthood [91].

## Conclusions

In summary, this neuropsychological study of children and adolescents at HR for OCD identified impairments in spatial working memory and trend in significance for impairment in motor and processing speed when compared to NOC. Future longitudinal studies following children at HR for OCD are required to investigate cognitive dysfunction as a vulnerability marker for the disorder, which may enhance the prevention of OCD among children and adolescents.

## Supplementary information

**Additional file 1: Figure S1**. Flow chart - design and recruitment of High Risk and non-OCD controls.

## Data Availability

The datasets analyzed during the current study are available from the corresponding author on reasonable request.

## Abbreviations

CBTT: Corsi block-tapping task
DSM-IV: Diagnostic and Statistical Manual of Mental Disorder Fourth Edition
DST: Digit span test
EF: Executive function
FDRs: First-degree relatives
FSIQ: Full-Scale Intelligence Quotient
HR: High-risk
IQ: Intelligence Quotient
K-SADS-PL: Kiddie Schedule For Affective Disorders And Schizophrenia for School Aged-Children
MANOVA: Multivariate Analysis of Variance
NOC: Non-OCD controls
OC: Obsessive-compulsive
OCD: Obsessive-compulsive disorder
OCS: Obsessive-compulsive symptoms
PIQ: Performance Intelligence Quotient
SCID-I/P: Structured Clinical Interview for DSM-IV-TR Axis I Disorders, Patient Edition.
TMT: Trail making test
VIQ: Verbal Intelligence Quotient
Y-BOCS: Yale-Brown Obsessive-Compulsive Scale
