# Supplementary material for: Cognitive performance in children and adolescents at high-risk for obsessive-compulsive disorder
Source: BMC Psychiatry. 2020 Jul 20;20:380. doi: 10.1186/s12888-020-02751-5 (PMC7370498; doi:10.1186/s12888-020-02751-5)
Supplement: Supplementary file 1 — Additional file 1: Figure S1. Flow chart - design and recruitment of High Risk and non-OCD controls. [file 12888_2020_2751_MOESM1_ESM.docx]

Figure S1

***High Risk***

Siblings or offsprings of OCD patients

Screening telephone call

n=278 candidates

***Non-OCD controls***

Media advertisements and active search at private and public schools

n=60

*Excluded (n=240)*

History of head injury;

History of substance abuse;

Presence of intellectual disability or any other psychiatric diagnosis according to DSM-IV;

Presence of any neurological condition;

Pregnancy or lactation.

n=38

*Excluded (n=6)*

History of head injury;

History of substance abuse;

Presence of intellectual disability or any other psychiatric diagnosis according to DSM-IV;

Presence of any neurological condition;

Pregnancy or lactation;

First degree relative of a subject with OCD;

Present OCS.

n=54

Evaluated with K-SADS-PL and Y-BOCS

n=23

Evaluated with psychiatric and cognitive measures

n=42

*Did not complete any inclusion criteria (n=5)*

Absence of OCS n=2;

Fulfilling criteria for OCD n=3.

n=18

Selected by similar age, IQ, education level, handedness, and puberty development of High-Risk group

n=31

**Figure S1**. Flow chart - design and recruitment of High Risk and non-OCD controls
